# Statistical significance and publication reporting bias in abstracts of reproductive medicine studies

**DOI:** 10.1093/humrep/dead248

**Published:** 2023-11-28

**Authors:** Qian Feng, Ben W Mol, John P A Ioannidis, Wentao Li

**Affiliations:** Department of Obstetrics and Gynaecology, Monash University, Clayton, VIC, Australia; Department of Obstetrics and Gynaecology, Monash University, Clayton, VIC, Australia; Aberdeen Centre for Women’s Health Research, School of Medicine, Medical Sciences and Nutrition, University of Aberdeen, Aberdeen, UK; Department of Medicine, Stanford University, Stanford, CA, USA; Department of Epidemiology and Population Health, Stanford University, Stanford, CA, USA; Department of Biomedical Data Science, Stanford University, Stanford, CA, USA; Department of Statistics, Stanford University, Stanford, CA, USA; Meta-Research Innovation Center at Stanford (METRICS), Stanford University, Stanford, CA, USA; Department of Obstetrics and Gynaecology, Monash University, Clayton, VIC, Australia

**Keywords:** publication reporting bias, significance chasing, publication bias, reporting quality, *P*-values

## Abstract

**STUDY QUESTION:**

What were the frequency and temporal trends of reporting *P*-values and effect measures in the abstracts of reproductive medicine studies in 1990–2022, how were reported *P*-values distributed, and what proportion of articles that present with statistical inference reported statistically significant results, i.e. ‘positive’ results?

**SUMMARY ANSWER:**

Around one in six abstracts reported *P*-values alone without effect measures, while the prevalence of effect measures, whether reported alone or accompanied by *P*-values, has been increasing, especially in meta-analyses and randomized controlled trials (RCTs); the reported *P*-values were frequently observed around certain cut-off values, notably at 0.001, 0.01, or 0.05, and among abstracts present with statistical inference (i.e. *P*-value, CIs, or significant terms), a large majority (77%) reported at least one statistically significant finding.

**WHAT IS KNOWN ALREADY:**

Publishing or reporting only results that show a ‘positive’ finding causes bias in evaluating interventions and risk factors and may incur adverse health outcomes for patients.

Despite efforts to minimize publication reporting bias in medical research, it remains unclear whether the magnitude and patterns of the bias have changed over time.

**STUDY DESIGN, SIZE, DURATION:**

We studied abstracts of reproductive medicine studies from 1990 to 2022. The reproductive medicine studies were published in 23 first-quartile journals under the category of Obstetrics and Gynaecology and Reproductive Biology in Journal Citation Reports and 5 high-impact general medical journals (*The Journal of the American Medical Association*, *The Lancet*, *The BMJ*, *The New England Journal of Medicine*, and *PLoS Medicine*). Articles without abstracts, animal studies, and non-research articles, such as case reports or guidelines, were excluded.

**PARTICIPANTS/MATERIALS, SETTING, METHODS:**

Automated text-mining was used to extract three types of statistical significance reporting, including *P*-values, CIs, and text description. Meanwhile, abstracts were text-mined for the presence of effect size metrics and Bayes factors. Five hundred abstracts were randomly selected and manually checked for the accuracy of automatic text extraction. The extracted statistical significance information was then analysed for temporal trends and distribution in general as well as in subgroups of study designs and journals.

**MAIN RESULTS AND THE ROLE OF CHANCE:**

A total of 24 907 eligible reproductive medicine articles were identified from 170 739 screened articles published in 28 journals. The proportion of abstracts not reporting any statistical significance inference halved from 81% (95% CI, 76–84%) in 1990 to 40% (95% CI, 38–44%) in 2021, while reporting *P*-values alone remained relatively stable, at 15% (95% CI, 12–18%) in 1990 and 19% (95% CI, 16–22%) in 2021. By contrast, the proportion of abstracts reporting effect measures alone increased considerably from 4.1% (95% CI, 2.6–6.3%) in 1990 to 26% (95% CI, 23–29%) in 2021. Similarly, the proportion of abstracts reporting effect measures together with *P*-values showed substantial growth from 0.8% (95% CI, 0.3–2.2%) to 14% (95% CI, 12–17%) during the same timeframe. Of 30 182 statistical significance inferences, 56% (n = 17 077) conveyed statistical inferences via *P*-values alone, 30% (n = 8945) via text description alone such as significant or non-significant, 9.3% (n = 2820) via CIs alone, and 4.7% (n = 1340) via both CI and *P*-values. The reported *P*-values (n = 18 417), including both a continuum of *P*-values and dichotomized *P*-values, were frequently observed around common cut-off values such as 0.001 (20%), 0.05 (16%), and 0.01 (10%). Of the 13 200 reproductive medicine abstracts containing at least one statistical inference, 77% of abstracts made at least one statistically significant statement. Among articles that reported statistical inference, a decline in the proportion of making at least one statistically significant inference was only seen in RCTs, dropping from 71% (95% CI, 48–88%) in 1990 to 59% (95% CI, 42–73%) in 2021, whereas the proportion in the rest of study types remained almost constant over the years. Of abstracts that reported *P*-value, 87% (95% CI, 86–88%) reported at least one statistically significant *P*-value; it was 92% (95% CI, 82–97%) in 1990 and reached its peak at 97% (95% CI, 93–99%) in 2001 before declining to 81% (95% CI, 76–85%) in 2021.

**LIMITATIONS, REASONS FOR CAUTION:**

First, our analysis focused solely on reporting patterns in abstracts but not full-text papers; however, in principle, abstracts should include condensed impartial information and avoid selective reporting. Second, while we attempted to identify all types of statistical significance reporting, our text mining was not flawless. However, the manual assessment showed that inaccuracies were not frequent.

**WIDER IMPLICATIONS OF THE FINDINGS:**

There is a welcome trend that effect measures are increasingly reported in the abstracts of reproductive medicine studies, specifically in RCTs and meta-analyses. Publication reporting bias remains a major concern. Inflated estimates of interventions and risk factors could harm decisions built upon biased evidence, including clinical recommendations and planning of future research.

**STUDY FUNDING/COMPETING INTEREST(S):**

No funding was received for this study. B.W.M. is supported by an NHMRC Investigator grant (GNT1176437); B.W.M. reports research grants and travel support from Merck and consultancy from Merch and ObsEva. W.L. is supported by an NHMRC Investigator Grant (GNT2016729). Q.F. reports receiving a PhD scholarship from Merck. The other author has no conflict of interest to declare.

**TRIAL REGISTRATION NUMBER:**

N/A.

## Introduction

Running statistical tests is a widely used tool in the quest for answers in medical research. Oftentimes, however, research is oversimplified, or even degraded, into focusing exclusively on the dichotomy of whether these tests are statistically significant or not. This oversimplification can compel the researchers to seek results in favour of statistical significance, a practice commonly referred to as ‘significance chasing’. This is part of a bigger problem known as publication reporting bias, which reflects the tendency for authors, reviewers, and editors to submit, accept, and publish articles based on not only the quality of the research but also on the hypothesis tested, and the significance and direction of effects estimated. Selective publication and reporting according to whether the results were statistically significant, or synonymously, ‘positive’, is a common form of publication reporting bias (Dickersin, 1990). Some researchers erroneously equate ‘positive’ findings to the importance of a study and therefore strive to obtain statistically significant results when designing and conducting their work, or preferentially report or highlight the ‘positive’ results in the abstracts. Common practices of significance hunting in research include conducting analysis on dozens of variables and only reporting statistically significant results, splitting or grouping variables to find significant results, or wilfully removing or including outliers after the data analysis has begun.

The practice of significance hunting is further exacerbated by journals that preferably accept articles with ‘positive’ results, as they often yield more readership and higher citations than those with ‘negative’ findings (Olson *et al.*, 2002; Duyx *et al.*, 2017). Compared with articles that reported ‘negative’ results, articles with ‘positive’ findings are twice as likely to be published, have shorter submission-to-publication time, and are more likely to be accepted by journals with high impact factors (Hopewell *et al.*, 2009; Siontis *et al.*, 2011). These are all powerful incentives for reinforcing authors’ enthusiasm for chasing significance. One result of this self-perpetuating cycle is that evidence in biomedicine tends to be replete with striking ‘positive’ findings that are often not plausible (Kyzas *et al.*, 2007; Coronado-Montoya *et al.*, 2016); while the number of articles with ‘negative’ findings is disproportionally low, resulting in research waste and biasing secondary analyses and decisions built upon such evidence (Fanelli, 2012).

The presence and ills of these reporting biases were recognized as early as several decades ago (Chalmers *et al.*, 1990; Dickersin, 1990). Outcries against the problem and strategies to remedy the issue have been repeatedly raised with some being implemented (Moher *et al.*, 2001; Wasserstein and Lazar, 2016; Wasserstein *et al.*, 2019; Page *et al.*, 2021). Evaluating how frequently the presence of statistically significant results in literature has changed over time could provide information in understanding to what extent the magnitude of these biases has abated. An earlier study by Chavalarias *et al.* (2016) showed that among the *P*-value presented abstracts of biomedicine articles in 1990–2014, a preponderant proportion of them (96%) reports *P*-values lower than 0.05, with little change during this time period. Such abundance of the statistically significant *P*-value was also observed in other fields (Cristea and Ioannidis, 2018; To and Jukes, 2019). However, estimating the trend of statistical significance reporting by tallying the frequency of *P*-value lower than 0.05 means omitting statistically significant results expressed in statistical measures other than *P*-values. In particular, resonating with the generations of warnings against the misuse of *P*-values (Cohen, 1994; Piccirillo, 2016; Yaddanapudi, 2016), authors have been increasingly using CIs to describe results, sometimes in the absence of *P*-values. This preference towards CIs is because they indicate statistical significance as well as the precision of the estimate. They have higher informativeness than the mere notation of statistical significance. Increasing adoption of CIs means a glut of statistically significant CIs could have been missed if only looking at *P*-values. Such limitation is especially pronounced when analysing articles published in journals in which the use of the *P*-value is discouraged or banned entirely (Woolston, 2015). While CIs have desirable properties and can give a sense of the range and uncertainty for effect sizes, they may also be readily affected by significance chasing (e.g. seeking CIs that exclude the null).

In this article, we aim to investigate three issues in the field of reproductive medicine between 1990 and 2022: first, the trend of reporting statistical inference in the forms of *P*-value, effect measures and Bayes metrics in abstracts of articles published; second, the distribution of reported *P*-values; and third, the frequency of reporting at least one statistically significant finding in the abstracts of papers (as *P*-values, as CI excluding the null, and/or as a text statement).

## Materials and methods

This is a meta-epidemiological study of research articles in the field of reproductive medicine published between 1990 and 2022. The study protocol was made in advance and finalized before the start of the data extraction and is available at https://osf.io/j97ap.

### Identification of eligible reproductive medicine articles

We constructed a database by retrieving potential reproductive medicine articles published between 1 January 1990 and 25 November 2022. First, we retrieved all articles published in Quantile 1 (Q1) journals indexed in Journal Citation Reports (JCR) 2021 under the categories Reproductive Biology (7 journals) and Obstetrics and Gynaecology (16 journals); the former primarily publish reproductive medicine articles, while the latter publish reproductive medicine articles alongside other topics in women’s health. A complete list of journals can be found in Supplementary Table S1. Second, we identified articles about women’s health published in five high-impact general medicine journals, i.e. *The Lancet*, *The Journal of the American Medical Association*, *The New England Journal of Medicine*, *PLoS Medicine*, and *The BMJ.* These women health articles were identified using a combination of free-text words and Medical Subject Headings terms and the complete searching strategy is provided in the Supplementary Data File S1.

Since the retrieved articles included articles from reproductive medicine, but also all other fields of women’s health (gynaecology and obstetrics fields), we used a search strategy to tease out reproductive articles from the rest (Supplementary Data File S2). The strategy to identify reproductive medicine articles was adapted from the search strategy developed by the information specialist from the Cochrane Gynaecology and Fertility Group (Supplementary Data File S3).

Among the retrieved reproductive medicine articles, we excluded: articles without abstracts; commentaries, letters to the editor, editorials, case reports, case series, guidelines, consensus, committee opinions, retracted papers, and qualitative systemic reviews (unless a meta-analysis was part of the review); and articles in which the subjects of the study were not humans. All exclusions were applied by identifying the words that are specific to these exclusion criteria in the article’s title such as ‘case report’, ‘a committee opinion’, ‘macaque’, or ‘mice’. The strategy to tease out these studies is provided in Supplementary Data File S4.

### *P*-value and effect measures extraction

Automated text-mining was used to extract three types of statistical significance reporting including *P*-value, CI, and words describing statistical significance.

#### Extracting P-values

The *P*-values were extracted using automated text-mining in R (R Core Team, 2013, version 3.6.1). The automated *P*-value extraction query was adapted based on the algorithm developed by Chavalarias *et al.* (2016) (Supplementary Data File S5). In brief, we defined the *P*-value reporting by starting with ‘*p*’, ‘*P*’, ‘*P*-value(s)’, ‘*p* value(s)’, or ‘*P* for trend’, followed by an equality or inequality signs (e.g. =, >, or <) or the text words describing the equality or inequality (e.g. ‘higher than’, ‘less than’, or ‘equal to’) and ending with digits (e.g. 0.01, 0.05). The retrieved *P*-values that were above 1 were excluded because these *P*-values did not mean statistical significance (e.g. *P* stands for progesterone level), typos made by authors themselves, or due to extraction errors. Such exclusion made up less than 0.1% of all extracted *P*-values (16/18 433). In addition, *P*-values followed by signs of ‘≥’ or ‘≤’ were classified into ‘<’ or ‘>’, respectively; this re-classification only made up for 1.0% (192/18 417) of extracted *P*-values. *P*-value equals to or less than 0.05 was classified as statistically significant.

#### Extracting text describing the statistical significance

In sentences where neither *P*-value nor CI was present, we further identified the presence of terms indicating significance. We identified the words describing ‘not statistically significant’ or ‘statistically significant’. We defined and retrieved the three main types of expressions of statistical significance from abstracts:

- A is ‘statistically significantly higher (or above, lower, increased, etc.)’ than B.
- We found ‘no significant difference’ between A and B.
- We found ‘no difference’ between A and B.

Although the second description is ambiguous in whether the difference is ‘statistically’ or ‘clinically’ or ‘biologically’ significant, we assumed that author meant statistically significant in the abstract unless specified. Similarly, in the third description, we assumed the author meant for ‘no statistically significant difference’. We then did a sensitivity analysis by only including the first description that is unambiguous in statistical significance.

#### Extracting significance inference statistics other than P-value

We extracted measures of effect size such as odds ratios, Glass's delta, and Cohen’s d from abstracts, either reported alone or accompanied *P*-values. We also collected information on reporting of Bayes factors and related Bayesian statistics. The list of effect sizes was adapted from the Table 1.1 Common Effect Size Indexes in the book *The Essential Guide to Effect Sizes* by Ellis (2010); the R code to extract effect sizes, Bayes factors, and related Bayesian statistics are provided in Supplementary Data File S6.

We extracted CIs, which were defined as expressions starting with the word ‘confidence interval’ or ‘CI’, followed by paired numbers denoting its range. Meanwhile, the types of point estimates that preceded the CI, such as ‘odds ratio’, ‘mean difference’, or ‘risk ratio’, were extracted simultaneously for confirming the statistical significance of the corresponding CI.

Once the CI and their point estimate were extracted, we translated the CI to statistical significance inference based on its context using the following rules. If the point estimate is a mean difference between the two groups and its CI does not include zero, it was considered statistically significant, and vice versa. If the point estimate is risk ratio, odds ratio, or hazard ratio and 1.00 was not within its CI, it was considered statistically significant, and vice versa.

To understand the possible inconsistency between *P*-value and CI, we simultaneously extracted *P*-values and CIs in sentences that reported both. We then counted the cases where the reported *P*-values were statistically significant but the CIs suggested the opposite, or vice visa.

### Reporting style definition

We reported four types of reporting styles in statistical inferences: *P*-value alone, defined as reporting *P*-value without effect measures; effect measures alone, defined as reporting effect measures without *P*-values; both *P*-values and effect measures were reported; and neither *P*-values nor effect measures were reported. Of note, the effect measure is considered present as long as its point estimate is reported, irrespective of whether it is accompanied by a CI. The presence of text in describing statistical significance was not investigated when looking at the reporting styles but was investigated when studying the proportion of abstracts making at least one statistically significant statement.

### Subgroup definitions

We examined the trend of statistical significance reporting in two subgrouping analyses: by the journals in which the article was published and by the study designs of the study. For the latter subgroups, articles were categorized into randomized controlled trials (RCTs), observational studies, clinical trials (non-randomized), and meta-analyses based on the classification made by the National Center for Biotechnology Information (NCBI) for filtering articles in the PubMed webpage. Detailed definitions of each study design can be found on the NCBI website (NCBI, 2022). The classification in study design by NCBI was tested by other groups and it fared generally well (McKibbon *et al.*, 2009; Lee *et al.*, 2012). Since observational studies were not categorized by NCBI until 2013 and their strategy to identify observational studies is not available to the public, the subgroup analysis for observational studies only included those published after 2013. To understand the reporting pattern in the basic research, we constructed a search strategy using a combination of free-text words to identify basic research (Supplementary Data File S7).

### Manual verification

A total of 300 articles from the 146 189 excluded articles were randomly selected for the manual check to confirm whether they fulfilled the inclusion criteria but were mistakenly excluded. In addition, we randomly selected another 200 articles from 24 500 included articles to confirm the presence, accuracy, and context of statistical significance and effect measures extracted (Supplementary Data File S8). All manual checking was done by the first author. This manual check was performed for checking three types of information. The first was to check whether articles were mistakenly excluded or included by scanning their abstracts and titles. The second was to verify extractions on statistical inferences including *P*-values, CIs, and significance terms. For this part, genuine statistical inferences do not encompass a description of statistical significance cut-offs in the method section (e.g. ‘*P* < 0.05 was considered as statistically significant’) or statistical significance inferences for baseline characteristics (e.g. ‘all baseline characteristics were not statistically significant, *P* > 0.05’). The last part was to confirm whether basic research was correctly classified.

### Statistical analysis

We reported descriptive statistics using the number and proportion of abstracts. Two-sided CIs for the single proportion were calculated using the built-in command in R. Subgroup analyses were performed in different study designs and journals in which the articles were published. All decimals are reported to two significant figures. All analyses were performed in R (R Core Team, 2013, version 3.6.1).

## Results

**Figure 1.**
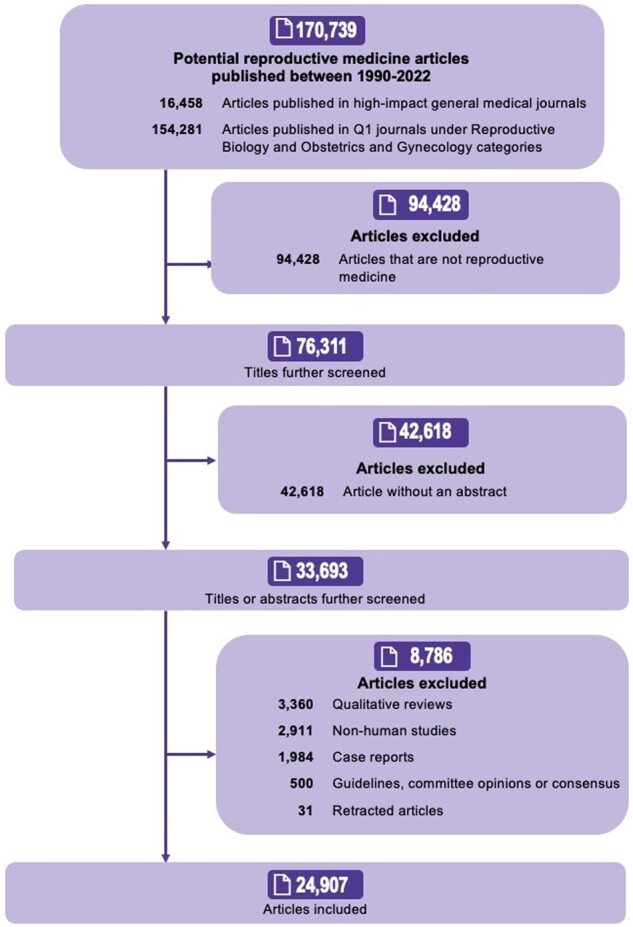
**Flowchart of the study of statistical significance and publication reporting bias in abstracts of reproductive medicine studies**.

Among 170 739 articles published in 28 selected journals, 76 311 reproductive medicine articles were identified, of which 33 693 (44%) included an abstract. After excluding 8786 articles that did not fulfil the inclusion criteria, a total of 24 907 articles were included. Figure 1 shows the flowchart of the study.

### Reporting *P*-values, effect measures, and Bayes factors over time

**Figure 2.**
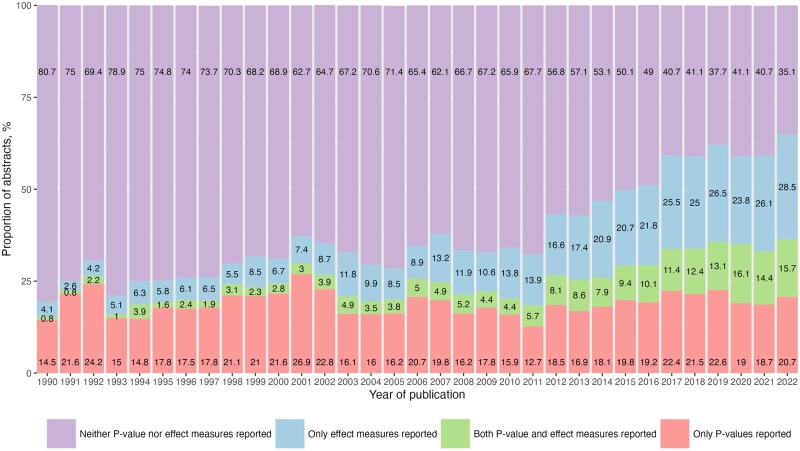
**The proportion of abstracts reporting *P*-value and/or effect measures in the abstract of reproductive medicine articles over time.** Only 19 abstracts mentioning Bayes factors were found in 24 907 articles. Owing to the trivial proportion, they were not given a separate label in the figure but were grouped with effect measures.

Overall, the proportion of abstracts reporting *P*-values alone remained relatively stable, which was 15% (95% CI, 12–18%) in 1990 and 19% (95% CI, 16–22%) in 2021 (Fig. 2). By contrast, the proportion of abstracts reporting effect measures alone surged from 4.1% (95% CI, 2.6–6.3%) in 1990 to 26% (95% CI, 23–29%) in 2021. Similarly, there was a remarkable growth in the proportion of abstracts reporting effect measures in combination with *P*-values, up from 0.8% (95% CI, 0.3–2.2%) to 14% (95% CI, 12–17%) during the same period. The proportion of abstracts neither reporting *P*-values nor effect measures halved from 81% (95% CI, 76–84%) to 40% (95% CI, 38–44%). Among 24 907 abstracts, only 19 were found to report Bayes factors or related Bayesian statistics.

**Figure 3.**
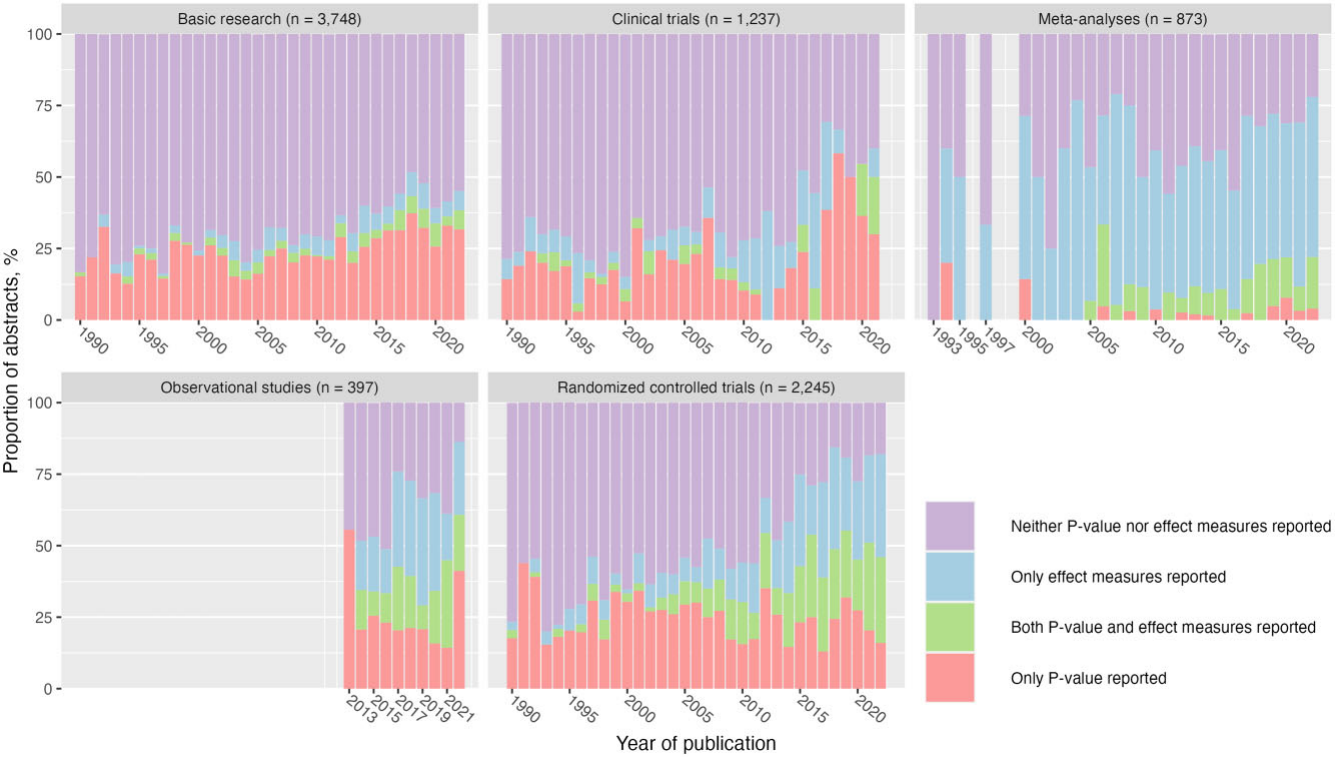
**The proportion of abstracts reporting *P*-value and/or effect measures in the abstract of reproductive medicine by study design.** The column before 2013 was not plotted in observational studies because the classification data was not provided by the National Institutes of Health. Some columns before 2000 were missing because no meta-analysis in reproductive medicine was found in these years.

In 2021, the proportion of abstracts reporting *P*-values alone was 3.2% (95% CI, 0.8–9.7%) in meta-analyses, 14% (95% CI, 6.4–28%) in observational studies, 20% (95% CI, 11–35%) in RCTs, 30% (95% CI, 8.1–65%) in clinical trials, and 33% (95% CI, 24–44%) in basic research (Fig. 3). Over time, the proportion of abstracts reporting *P*-values alone decreased in RCTs and meta-analyses but rose mildly in basic research.

Regarding the proportion of abstracts reporting effect measures alone in 2021, it was 5.3% (95% CI, 2.0–13%) in basic research, 10% (95% CI, 0.5–46%) in clinical trials, 16% (95% CI, 7.8–30%) in observational studies, 31% (95% CI, 19–46%) in RCTs, and 57% (95% CI, 47–68%) in meta-analyses. RCTs saw a steady increase in the proportion of reporting effect measures since roughly 2010 while the proportion remained unchanged for the rest of study designs.

Over time, the proportion of abstracts using both *P*-values and effect measures rose from 2.9% (95% CI, 0.2–17%) in 1990 to 31% (95% CI, 19–46%) in 2021 in RCTs. By contrast, the proportion of abstracts using both *P*-value and effect measures stayed merely unchanged in the basic research, at the low of 3.2% (95% CI, 0.8–9.7%) in 2021.

**Figure 4.**
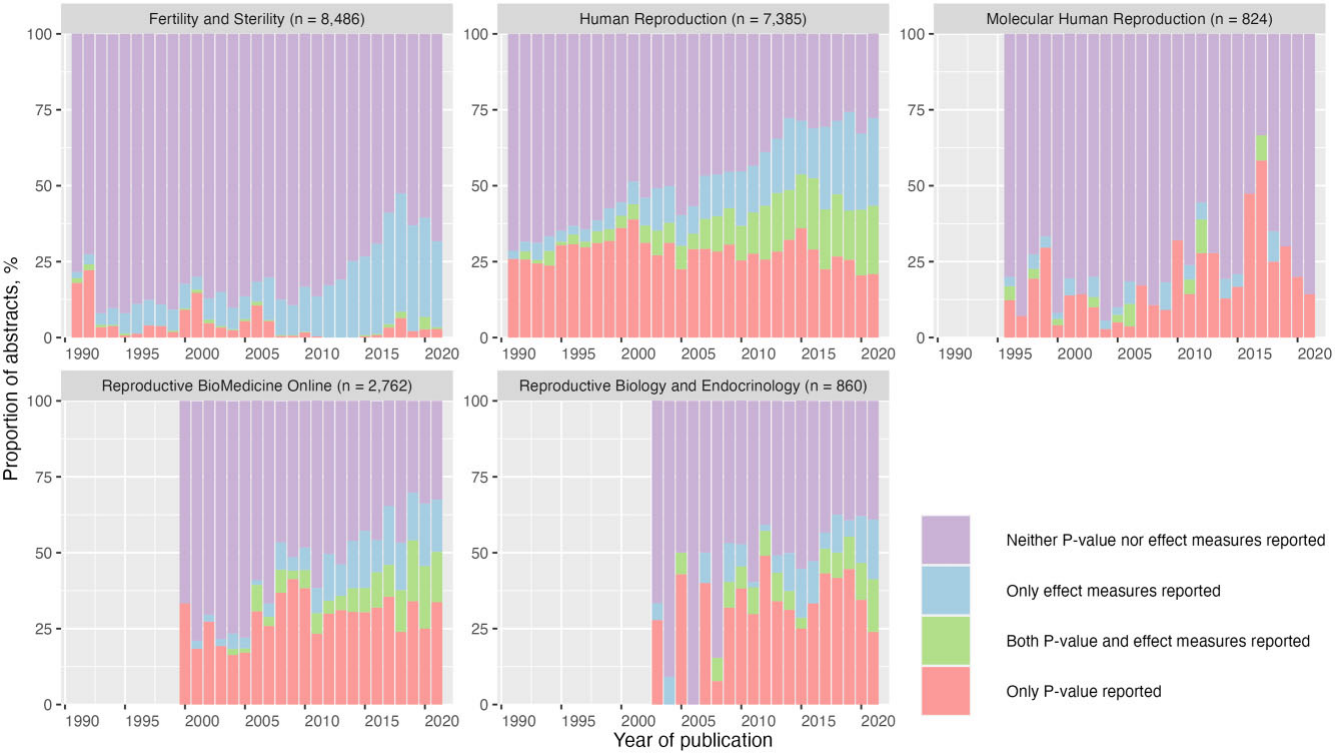
**The proportion of abstracts reporting *P*-value and/or effect measures in the abstract of reproductive medicine articles by selected journals.** The column before certain years is missing because the journal was not founded before that time point.

In terms of the proportion of abstracts reporting *P*-value alone by journal in 2021 (Fig. 4), it was 2.8% (95% CI, 1.0–6.7%) in *Fertility and Sterility*, 14% (95% CI, 2.5–44%) in *Molecular Human Reproduction*, 21% (95% CI, 15–28%) in *Human Reproduction*, 24% (95% CI, 16–34%) in *Reproductive Biology and Endocrinology*, and 34% (95% CI, 27–42%) in *Reproductive Biomedicine Online*. In *Fertility and Sterility*, the proportion of abstracts adopting effect measures alone increased from 3.2% (95% CI, 1.5–6.4%) in 1990 to 28% (95% CI, 22–36%) in 2021. Likewise, a sharp growth in reporting effect measures alone was seen in *Human Reproduction*, up from 4.7% (95% CI, 1.9–10%) in 1990 to 29% (95% CI, 23–36%) in 2021. There had been a gradual increase in the proportion of abstracts using both *P*-value and effect measures in all journals except for *Molecular Human Reproduction* and *Fertility and Sterility*. Within each journal, reporting patterns by study designs are provided in Supplementary Fig. S1; the results showed the reporting pattern is similar across different study designs.

### Distribution of reported *P*-values in abstracts

There were 30 182 statistical inferences (i.e. the sum of the number of *P*-values, CIs, and significance terms) described in 53% (13 200/24 907) of abstracts. Of them, 56% (n = 17 077) used *P*-values alone to convey statistical inference, 30% (n = 8945) used significance terms only, 9.3% (n = 2820) used CIs only, and 4.7% (n = 1340) used both *P*-value and CIs.

**Figure 5.**
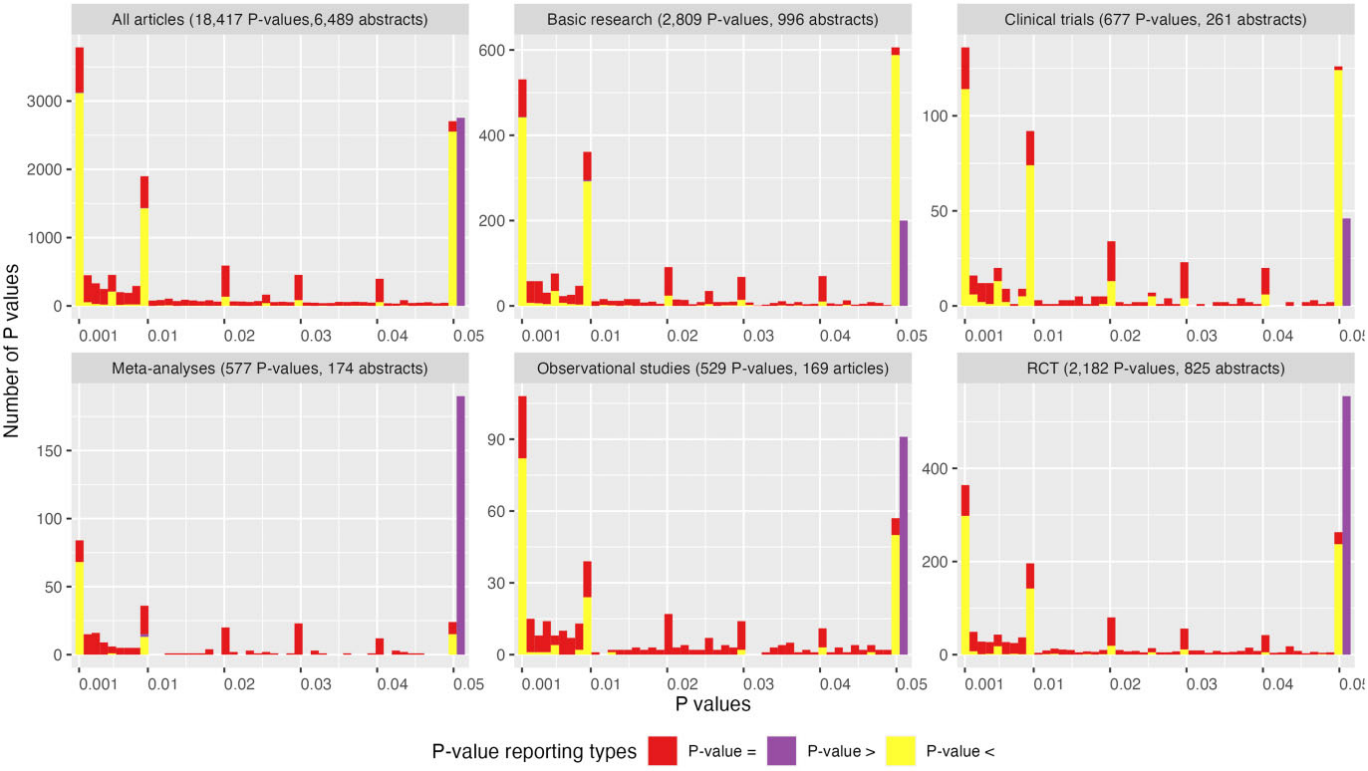
**The distribution of directly reported *P*-values in different study designs.** The *P*-value plotted in this figure only includes directly reported *P*-values, meaning inferred *P*-values converted from CIs or texts are not presented here. *P*-values reported over 0.05 (e.g. *P* > 0.2) were lumped together and plotted on a single bar at the *P*-value of 0.05 rather than plotting the exact value to avoid the long tail in the *X*-axis.

The reported *P*-values were frequently observed at a *P*-value of 0.001 (n = 3647, 20%), 0.05 (n = 3015, 16%), and 0.01 (n = 1889, 10%) (Fig. 5). Of 18 417 reported *P*-values, 37% were reported as a precise *P*-value, while the remaining reported *P*-values in a threshold. The percentage of extremely small *P*-values, defined as *P*-values ≤0.001, among all reported *P*-values was 29% (n = 5238). The proportion of extremely small *P*-values was 23% (491/2182) in RCTs, 27% (142/529) in observational studies, 27% (184/677) in clinical trials, 28% (773/2809) in basic research, and 31% (178/577) in meta-analyses. Subgroup analysis of *P*-value distribution among journals showed that the proportion of extremely small *P*-value was the highest in *Reproductive Biomedicine Online* at 32% (1001/3144) and the lowest in *Molecular Human Reproduction* at 23% (97/414) (Supplementary Fig. S2). The graph showing a full spectrum of extracted *P*-values indicates *P*-values heavily skewed to small values, irrespective of study type (Supplementary Fig. S3).

### Frequency of reporting statistically significant statement

**Figure 6.**
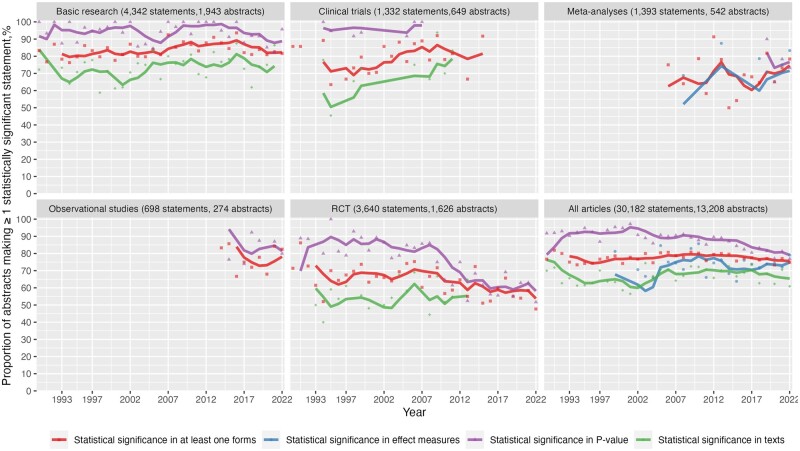
**Frequency of abstracts making ≥1 statistically significant statement among abstracts making at least one statistical inference by study design.** Four lines of different colours were not always present or continuous because if the total number of publications in a single year was less than six, it was not depicted. This was to show the overall trend and thus avoid the huge variations caused by a small number in a single year. The lines represent the rolling average of average proportion for 4 consecutive years, while the dots represent the exact proportion.

Of 13 200 abstracts reporting statistical significance inference (i.e. either in *P*-value, CIs, or significance terms), 77% (95% CI 76–78%) of them contain at least one statistically significant inference (Fig. 6). The proportion in abstracts that used *P*-value, CI, and text description was 87% (95% CI, 86–88%), 73% (95% CI, 70–76%), and 67% (95% CI, 66–69%), respectively. We further excluded articles that were ambiguous in describing their statistical meaning in texts. Among 1017 articles describing statistical significance in text unambiguously, 64% (95% CI 61–67%) reported at least one statistically significant result (Supplementary Fig. S4).

Among abstracts present with statistical significance inference, the proportion of having at least one statistically significant inference fluctuated at the beginning of the 1990s and then climbed gradually to 2009 at 81% (95% CI 77–84%) before decreasing to 77% (95% CI 73–81%). Similarly, among abstracts that reported at least one *P*-value, the proportion of abstracts with at least one statistically significant *P*-value went up gradually from 92% (95% CI, 82–97%) in 1990 and peaked at 97% (95% CI 93–99%) in 2001 before declining to 81% (95% CI, 76–85%) in 2021.

Over time, the decline in the proportion of making at least one statistically significant inference was only seen in RCTs, dropping from 71% (95% CI, 48–88%) in 1990 to 59% (95% CI, 42–73%) in 2021, whereas the proportion in the rest of study types remained almost constant over the years. Among reproductive medicine journals, *Human Reproduction* was the only journal with a reduction in the proportion of having at least one statistically significant *P*-value in abstracts that used *P*-values, down from 92% (95% CI, 81–97%) in 1990 to 84% (95% CI, 79–88%) in 2021, while the proportion remained constant in other journals (Supplementary Fig. S5).

In an estimate of inconsistency in significance inference between concurrently reported *P*-value and CI, 2.9% (39/1334) of pairs led to opposite conclusions (Supplementary Data File S9).

### Manual verification

The detailed manual check results are provided in Supplementary Data File S10. In brief, the sensitivity and specificity for extracting the *P*-value were 96% and 99%, respectively. The sensitivity and specificity for extracting CIs were 63% and 100%, respectively. The sensitivity for extracting significance terms was 67%, and the specificity was 90%. A sensitivity analysis in 200 manually verified abstracts showed the proportions containing at least one statistically significant finding in the form of CIs and texts were 83% and 81%, respectively.

## Discussion

On analysis of the statistical significance reporting in abstracts of reproductive medicine studies from 1990 to 2022, we found that reporting of *P*-values alone prevailed and did not abate over time, particularly in basic research. Encouragingly, effect measures, reported alone or accompanied by *P*-values, increased steadily, especially in RCTs and meta-analyses. *P*-values exhibited a strong tendency to dichotomize around certain cut-off values. While the proportion of abstracts reporting at least one statistically significant finding was unreasonably high, the proportion has been falling since 2000. Amidst this trend, RCTs stood out as the only study type showing a remarkable reduction in the proportion of abstracts reporting statistically significant results.

In comparison with previous studies using the same methodology, we included various types of statistical statements in the analysis, and not merely *P*-values, thereby providing a more inclusive portrait of reporting trend of statistical inference. Furthermore, we were able to stratify the results by study design, allowing us to probe the variations with respect to study type. Our study also has limitations. The first is that the textual corpus analysed is abstracts but not full-text articles because of the large number of articles and the fact that automated text-mining is less accurate in full-text. Abstracts indicate what authors wish to highlight, likely to be more selective than the results reported in the full text. However, selective reporting and overly emphasizing statistically significant results in the abstract is a form of reporting bias that has profound implications. Another limitation is that while we optimized the text-mining algorithm attempting to identify all types of statistical significance reporting, there were expressions that we incorrectly omitted. However, our manual assessment suggested that inaccuracies were not frequent and unlikely to affect the overall findings. Sensitivity estimates suggest that more CIs and verbal significance statements were missed than *P*-values. These inaccuracies are intrinsic to text-mining methods when grasping semantic meaning through text patterns as human language is hugely diverse and the meaning of the words varies according to the context. Finally, there might have been imprecision when interpreting statistical inference in the following scenarios. Some researchers focus primarily on estimating effects without engaging in null hypothesis significance testing; *P*-values, CIs, or texts describing statistical significance could have been used for baseline descriptive results and did not denote the rejection or acceptance of the null hypothesis; and 0.05 is not universally applied across all research contexts as the Type I error rate. However, given the widespread use of null hypothesis significance testing in which the Type I error rate is often set as 0.05, we believe these imprecise estimations are infrequent and do not considerably affect our results.

The causes behind the persisting reliance on *P*-value alone reporting are various. One is that null hypothesis significance testing, from which *P*-values are derived, is deeply ingrained in the statistic curriculum and practice (Stang *et al.*, 2021), while tools to complement *P*-values, such as effect sizes, or alternatives to null hypothesis significance testing, such as Bayesian factors, receive far less attention (Karadaghy *et al.*, 2017; Ioannidis, 2019). Despite some journals discouraging authors from reporting *P*-value alone or banning *P*-value outright at the submission stage (Woolston, 2015), to the best of our knowledge, only a few of the journals included in our analysis did so (Harrington *et al.*, 2019; Human Reproduction, 2023; JAMA, 2023). This leads to a substantial number of articles with *P*-value alone getting published, entrenching the reader’s impression that such reporting is adequate. However, reducing the results into a binary conclusion of ‘significant’ or ‘not significant’ could be misleading; this applies not only to reported *P*-values, but even more so when verbal statements are made about statistical significance (or absence) with neither *P*-values nor effect sizes. Of note, while *P*-values inform readers of the incompatibility between the dataset and the specified statistical model, they do not give information on the size of the effect, preventing the readers from determining whether the effect is biologically or clinically relevant (Wasserstein and Lazar, 2016). Solitary verbal statements are even less informative.

We observed a growing predilection for reporting effect measures alone or accompanied by *P*-value over time, a welcome trend driven by multiple forces working in tandem. There have been repeated calls from statisticians advocating effect measures reporting (Braitman, 1988; Wasserstein and Lazar, 2016; Wasserstein *et al.*, 2019), propelling authors to change their reporting practices and journals to update their author’s guide—and some have done so (Harrington *et al.*, 2019). Our observation of vast variations in the prevalence of effect measures among journals highlights the possible impact of journals’ position on reporting patterns. For example, in journals in which the author’s guide contains explicit recommendations for reporting effect measures, such as *Fertility and Sterility* and *Human Reproduction*, reporting effect measures was more common (Fertility and Sterility, 2023; Human Reproduction, 2023), while in journals that have no such recommendation effect measures uptake was generally low (Wayant *et al.*, 2018; Reproductive Biomedicine Online, 2023). However, it is also likely that a higher proportion of effect measures uptake in some journals is due to their highly ranked status, which allows them to attract and publish articles that are written in higher quality and by teams that are more knowledgeable. Another contributing factor to the increasing uptake of effect measures is that standardized reporting checklists were made available early and were strictly adhered to in some areas. Such a case in point is RCTs, which exhibited remarkable growth in the proportion of abstracts using effect measures since early 2000s, roughly the time when CONSORTS (Consolidated Standards of Reporting Trials) was introduced and then increasingly endorsed by journals (Begg *et al.*, 1996; Moher *et al.*, 2001; Hopewell *et al.*, 2008). By contrast, in realms where standardized research reporting was not widely endorsed, such as basic research (Gallo *et al.*, 2012), we observed that *P*-values alone reporting was ubiquitous and effect measures were underused. This contrast between study types demonstrated the importance of making, endorsing, and implementing standardized reporting.

The frequent observation of *P*-values at 0.01 or 0.05 is in line with previous studies in other fields or general medicine (Chavalarias *et al.*, 2016). The explanation for its high frequency is probably a conventional use of the cut-off *P*-value in hypothesis testing and researchers hunt for statistically significant results by rounding or adjusting *P*-values to match these cut-offs. We also found an alarmingly high proportion of articles reported significant findings in abstracts, whether they were expressed in *P*-values, CIs, or text statements. The explanation of this phenomenon may lie in the academic reward system, where publishing ‘positive’ results is incentivized, and ‘negative’ is either unnoticed or disfavoured. Such inflated results could mislead the direction of future science and virtually all decisions translated from the evidence, ranging from patient care, research initiatives, and public health efforts to commercial investment.

Aside from significance hunting in the form of honest errors, such as parallel testing, post hoc data exploration, or selective reporting of ‘positive’ results, another important yet often overlooked factor contributing to the predominance of ‘positive’ findings is research misconduct through data fabrication or falsification. Data falsification accounts for one-fifth of retractions in the gynaecology and obstetrics fields (Chambers *et al.*, 2019). Among a sample of 33 retracted RCTs owing to questionable data validity in gynaecology and obstetrics, only 3 reported null findings (Li *et al.*, 2022); another study found none of the 14 RCTs retracted in obstetrics had reported null findings (Anderson *et al.*, 2023). Although the retractions are not many, such a low proportion of ‘negative’ findings among retractions implies a tendency for data fabricators to manipulate results in favour of positive ones to enhance the likelihood of their work being published. Considering data fraud that eventually led to retractions is only the tip of the iceberg, it remains unknown to what extent such research misconduct has inflated the prevalence of statistically significant findings (Li *et al.*, 2022).

On the other hand, we documented the proportion of ‘negative’ articles appears to be increasing slightly in recent years, and such a trend is more prominent in the form of *P*-value. This finding is consistent with previous studies and it could be that the efforts made in curbing the publication reporting bias had worked, albeit modestly (Chavalarias *et al.*, 2016; To and Jukes, 2019). Importantly, the remarkable decline in the proportion of studies with ‘positive’ results in RCTs indicates that mandated practices in this field, such as pre-registration of protocols and analysis plans made a priori required by some journals, may be partly stemming the bias. These practices have not become the standards for basic research and observational studies (Wadman, 2013; Braillon and Naudet, 2023), in which abstracts with ‘positive’ results remain a substantial majority. While emulating the measures taken in the RCTs could be a great lesson for the field overall, it remains unknown whether spin, defined as the use of specific reporting strategies to distort the interpretation of non-significant results to be more favourable, was part of those non-significant studies (Arunachalam *et al.*, 2017). Moreover, there is an increasing range of circumstances where non-significant results may be desirable (e.g. non-inferiority studies, or drug toxicity studies) (Ioannidis, 2023). Ultimately, the information value of the research should not be based on statistical significance alone, but rather the importance of the research question and the quality of the methods applied.

Because *P*-value is often misinterpreted as practical importance and better known than effect measures, there have been proposals of replacing all *P*-values with CIs to dampen the enthusiasm of tweaking *P*-values to chase significance (Gelman and Carlin, 2017; Gelman and Greenland, 2019). However, our findings indicate that while the proportion of articles with statistically significant findings expressed using CI is lower than that of using *P*-values, the majority of the findings were still statistically significant. Therefore, substituting all *P*-values with CIs alone may not be the solution to the rigid dichotomous thinking surrounding statistical significance and significance hunting.

Interestingly, we also observed that when results are statistically significant, authors prefer using *P*-values over verbal statements to report findings. This preference stems from the conventional practice of reporting ‘*P *<* *0.05’ for statistically significant findings. It is also a result of the popular misconception of equating the statistical significance to the importance of the study, propelling authors to include *P*-values to highlight the ‘importance’ of their work. By the same token, when results are not statistically significant, researchers often shun reporting *P*-values and simply use verbal statements; the belief behind this practice is that verbal statements might play down the presence of ‘negative findings’, while including ‘*P *>* *0.05’ would have made the work look less appealing to journals and readers.

## Conclusion

Reporting *P*-values alone remains enduringly frequent in abstracts of reproductive medicine articles, preventing readers from understanding results correctly. Encouragingly, there has been a positive shift towards using effect measures more frequently to convey statistical significance in abstracts. *P*-values dichotomizing at cut-off values of statistical significance appear to be prevalent and a majority of the abstracts reported statistically significant results, indicating publication reporting bias remains a concern in reproductive medicine literature. Such inflated treatment effects could mislead all types of patient care, plan of future research, and policy decisions. Nevertheless, there was a remarkable reduction in publication reporting bias in RCTs, serving as a valuable lesson in curbing the bias for other research types.

## Supplementary Material

dead248_Supplementary_Data_File_S1

dead248_Supplementary_Data_File_S2

dead248_Supplementary_Data_File_S3

dead248_Supplementary_Data_File_S4

dead248_Supplementary_Data_File_S5

dead248_Supplementary_Data_File_S6

dead248_Supplementary_Data_File_S7

dead248_Supplementary_Data_File_S8

dead248_Supplementary_Data_File_S9

dead248_Supplementary_Data_File_S10

dead248_Supplementary_Figure_S1

dead248_Supplementary_Figure_S2

dead248_Supplementary_Figure_S3

dead248_Supplementary_Figure_S4

dead248_Supplementary_Figure_S5

dead248_Supplementary_Table_S1

## Data Availability

The data underlying this article will be shared on reasonable request to the corresponding author.
