# Supplementary material for: Statistical significance and publication reporting bias in abstracts of reproductive medicine studies
Source: Hum Reprod. 2023 Nov 28;39(3):548–58. doi: 10.1093/humrep/dead248 (PMC10905502; doi:10.1093/humrep/dead248)
Supplement: dead248_Supplementary_Data_File_S1 [file dead248_supplementary_data_file_s1.pdf]

OXFORD human reproduction

Human Reproduction, 2023, 1–1
https://doi.org/10.1093/humrep/dead248
Supplementary Data

Supplementary Data File S1

The search strategy to identify women health studies among top general medicine journals.

NEJM

esearch -db pubmed -query ‘genital diseases, female[mh] OR infertility, female [mh] OR contraceptive devices, female [mh] OR reproductive control agents [mh] OR dysmenorrhea [mh] OR women s health [mh] OR pregnancy complications [mh] OR tuberculosis, female genital [mh] OR reproductive techniques [mh] OR reproduction [mh] AND The New England journal of medicine [Journal]’ -datetype PDAT -mindate 1990/01/01 -maxdate 2022/11/25 | efetch -format xml | xtract -pattern PubmedArticle -tab ‘|’ -sep ‘;’ -element MedlineCitation/PMID Journal/ISSN ISOAbbreviation PubDate/Year PublicationTypeList/Publication Type Language ArticleTitle -def ‘N/A’ -element Abstract/AbstractText -block Author -position last -element Author/ForeName Author/LastName AffiliationInfo/Affiliation | > NEJM1_last_author.csv

BMJ

esearch -db pubmed -query ‘genital diseases, female[mh] OR infertility, female [mh] OR contraceptive devices, female [mh] OR reproductive control agents [mh] OR dysmenorrhea [mh] OR women s health [mh] OR pregnancy complications [mh] OR tuberculosis, female genital [mh] OR reproductive techniques [mh] OR reproduction [mh] AND British medical journal[Journal]’ -datetype PDAT -mindate 1990/01/01 -maxdate 2022/11/25 | efetch -format xml | xtract -pattern PubmedArticle -tab ‘|’ -sep ‘;’ -element MedlineCitation/PMID Journal/ISSN ISOAbbreviation PubDate/Year PublicationTypeList/PublicationType Language ArticleTitle -def ‘N/A’ -element Abstract/AbstractText -block Author -position last -element Author/ForeName Author/LastName AffiliationInfo/Affiliation | > BMJ1_last_author.csv

JAMA

esearch -db pubmed -query ‘genital diseases, female[mh] OR infertility, female [mh] OR contraceptive devices, female [mh] OR reproductive control agents [mh] OR dysmenorrhea [mh] OR women s health [mh] OR pregnancy complications [mh] OR tuberculosis, female genital [mh] OR reproductive techniques [mh] OR reproduction [mh] AND JAMA[Journal]’ -datetype PDAT -mindate 1990/01/01 -maxdate 2022/11/25 | efetch -format xml | xtract -pattern PubmedArticle -tab ‘|’ -sep ‘;’ -element MedlineCitation/PMID Journal/ISSN ISOAbbreviation PubDate/Year PublicationTypeList/PublicationType Language ArticleTitle -def ‘N/A’ -element Abstract/AbstractText -block Author -position last -element Author/ForeName Author/LastName AffiliationInfo/Affiliation | > JAMA1_last_author.csv

Lancet

esearch -db pubmed -query ‘genital diseases, female[mh] OR infertility, female [mh] OR contraceptive devices, female [mh] OR reproductive control agents [mh] OR dysmenorrhea [mh] OR women s health [mh] OR pregnancy complications [mh] OR tuberculosis, female genital [mh] OR reproductive techniques [mh] OR reproduction [mh] AND Lancet[Journal]’ -datetype PDAT -mindate 1990/01/01 -maxdate 2022/11/25 | efetch -format xml | xtract -pattern PubmedArticle -tab ‘|’ -sep ‘;’ -element MedlineCitation/PMID Journal/ISSN ISOAbbreviation PubDate/Year PublicationTypeList/PublicationType Language ArticleTitle -def ‘N/A’ -element Abstract/AbstractText -block Author -position last -element Author/ForeName Author/LastName AffiliationInfo/Affiliation | > lancet1_last_author.csv

PLoS Medicine

esearch -db pubmed -query ‘genital diseases, female[mh] OR infertility, female [mh] OR contraceptive devices, female [mh] OR reproductive control agents [mh] OR dysmenorrhea [mh] OR women s health [mh] OR pregnancy complications [mh] OR tuberculosis, female genital [mh] OR reproductive techniques [mh] OR reproduction [mh] AND plos medicine[Journal]’ -datetype PDAT -mindate 1990/01/01 -maxdate 2022/11/25 | efetch -format xml | xtract -pattern PubmedArticle -tab ‘|-sep ‘;’ -element MedlineCitation/PMID Journal/ISSN ISOAbbreviation PubDate/Year PublicationTypeList/PublicationType Language ArticleTitle -def ‘N/A’ -element Abstract/AbstractText -block Author -position last -element Author/ForeName Author/LastName AffiliationInfo/Affiliation | > plosmed1_last_author.csv
