# Supplementary material for: Statistical significance and publication reporting bias in abstracts of reproductive medicine studies
Source: Hum Reprod. 2023 Nov 28;39(3):548–58. doi: 10.1093/humrep/dead248 (PMC10905502; doi:10.1093/humrep/dead248)
Supplement: dead248_Supplementary_Data_File_S2 [file dead248_supplementary_data_file_s2.docx]

# **Supplementary Data File S2** The R code to identify reproductive medicine articles.

**The free-text words to identify reproductive medicine article**

Repro_regex<-"[Aa]ntagonist\\sprotocol|[Aa]gonist\\sprotocol|[Zz]ona|[Ll]uteal\\sphase\\ssupport|[Ff]ecund|[iI]nfertility|[iI]nfertile|[Ff]ertil|[Ss]ub.?[Ff]ertility|[Ss]ub.?[Ff]ertile|[Rr]eproduct|[cC]onception[s]?|[zZ]ygote[s]?|[Gg]amete[s]?|[Bb]lastocyst[s]?|[sS]emen|[sS]perm[s]?|[Ee]mbryo[s]?|[Ss]eminal|[Oo]ocyte[s]?|[fF]ollic|[Oo]vulat|[eE]gg[s]?|[iI]n.?[vV]itro\\s?[mM]aturation|[fF]ertilization|[fF]ertilisation|[Ii]nsemination|[vV]itrif|[Cc]ryopreserv|[iI]ntracytoplasmic.?[sS]perm|([Oo]varian(\\s)?)?(hyper)+(.)?[sS]timulation|[Oo]varian\\s[Ss]timulation|[Ee]ndometrial.[pP]repration|[Ii]mplantation.?failure|[[Pp]re]?.?[iI]mplantation|[[Pp]eri]?.?mplantation|[Ee]ndometrial.?receptivity|[Ee]ndometrium.?receptivity|[Ff]ollicle.?[sS]timulating.?[hH]ormone[s]?|[Ll]uteinizing.?[hH]ormone[s]?|[Aa]nti.?m.?llerian.?hormone[s]?|(?i)IVF|(?i)IVM|(?i)ICSI|(?i)PG[tsdm]|(?i)IUI|(?-i)\bOI\b|\b(?-i)FET[\\s|-]?|(?-i)[\\s|-]ET\\s?|(?i)FSH|L[Hh]|\b(?-i)ERA\b|(?i)AMH|\b(?-i)ART\b|[tT]rophectoderm|[Oo]oogenesis|[aA]ntral.?[fF]ollicul|\b[Gg]onadotropin(s)?\b|[Cc]umulus|[Ee]ndometrial.?[Ss]cratch|([Ll]ow|[Gg]ood|[Pp]oor|([Ss]ub(\\s|-))?[Oo]ptimal)(\\s)?([Oo]varian)?(\\s)?[Rr]espon|[Tt]rophoblast|trigger|[Oo]hss|OHSS"

**The mesh words to detect reproductive medicine articles**

Repro_regex_mesh<-"[fF]ertilization|[fF]ertilisation|[Rr]eproduction|[Rr]eproductive|Infertility|Fertility|Preconception|Donor\\sConception|Embryo\\sTransfer|Embryo\\sImplantation|Cryopreservation|Oocyte[s]?|Semen|Sperm[s]|Seminal|Follicle Stimulating|Anti-Mullerian Hormone|Anovulation|Ovulation"
