# Supplementary material for: Statistical significance and publication reporting bias in abstracts of reproductive medicine studies
Source: Hum Reprod. 2023 Nov 28;39(3):548–58. doi: 10.1093/humrep/dead248 (PMC10905502; doi:10.1093/humrep/dead248)
Supplement: dead248_Supplementary_Data_File_S3 [file dead248_supplementary_data_file_s3.pdf]

OXFORD
human reproduction
Human Reproduction, 2023, 1–1
https://doi.org/10.1093/humrep/dead248
Supplementary Data

Supplementary Data File S3

The search strategy developed by the Cochrane Gynaecology and Fertility Group to identify infertility articles.

Date search strategy updated: 02.08.11

The strategy for identifying possible RCTs (terms 1–11) is the Cochrane sensitivity and precision maximising version devised by UK Cochrane Centre (see Cochrane Handbook Chapter 6, Box 6.4d). The strategy for identifying records within the scope of the CGFG was developed by the Information Specialist and the Co-ordinating Editor.

1. randomized controlled trial.pt.
2. controlled clinical trial.pt.
3. randomized.ab
4. placebo.tw
5. clinical trials as topic.sh
6. randomly.ab
7. trial.ti
8. (crossover or cross-over or cross over).ab
9. or/1-8
10. exp animals/not humans.sh
11. 9 not 10
12. exp reproductive techniques/
13. exp fertility agents/
14. (in vitro adj5 fertili$).tw.
15. ivf.tw.
16. icsi.tw.
17. (intracytoplas$ adj5 sperm$).tw.
18. subfertil$.tw.
19. (sperm$ adj5 inject$).tw.
20. suzi.tw.
21. (subzon$ adj5 sperm$).tw.
22. (zona adj5 dissect$).tw.
23. pzd.tw.
24. (ovar$ adj5 hyperstim$).tw.
25. ohss.tw.
26. (oocyt$ adj5 retriev$).tw.
27. (oocyt$ adj5 pickup).tw.
28. (sperm$ adj5 prepa$).tw.
29. (acrosom$ adj5 sperm$).tw.
30. (sperm$ adj5 stimul$).tw.
31. (sperm$ adj5 pentox$).tw.
32. (sperm$ adj5 caff$).tw.
33. (sperm$ adj5 kalli$).tw.
34. (sperm$ adj5 swimup$).tw.
35. (sperm adj5 percol$).tw.
36. (embry$ adj5 biops$).tw.
37. (cocult$ adj5 embry$).tw.
38. (cocult$ adj5 trophobl$).tw.
39. (luteal phase adj5 support).tw.
40. (sperm$ adj5 cryopreserv$).tw.
41. (frozen adj5 embry$).tw.
42. (antisperm$ adj5 antibod$).tw.
43. (sperm$ adj5 antibod$).tw.
44. (artific$ adj5 inseminat$).tw.
45. (cervi$ adj5 inseminat$).tw.
46. (fallopian tub$ adj5 perfus$).tw.
47. (luteal adj5 defect$).tw.
48. (luteal adj5 dysfunct$).tw.
49. (ovulat$ adj5 induc$).tw.
50. (intraut$ adj5 inseminat$).tw.
51. (ectop$ adj5 preg$).tw.
52. (tub$ adj5 preg$).tw.
53. inseminat$.tw.
54. varicoc$.tw.
55. fertility/
56. fertilization/
57. insemination/
58. ¬embryo implantation/
59. Ovarian Hyperstimulation Syndrome/
60. (embry$ adj5 transf$).tw.
61. or/12-60
62. ¬11 and 61
63. *Breeding/
64. *Consanguinity/
65. (plant$ adj5 hybridi$).tw.
66. *plants/ or *plants, edible/ or *power plants/
67. or/63-66
68. ¬62 not 67
69. limit¬68 to latest update

Embase (OVID Online updated weekly) Years covered: 1980—ongoing

Date search strategy updated: 02.08.11.

Notes: the above is available and was downloaded from https://cgf.cochrane.org/sites/cgf.cochrane.org/files/public/uploads/uploads/search_strategies_for_the_identification_of_studies_cgf.pdf
