# Supplementary material for: Statistical significance and publication reporting bias in abstracts of reproductive medicine studies
Source: Hum Reprod. 2023 Nov 28;39(3):548–58. doi: 10.1093/humrep/dead248 (PMC10905502; doi:10.1093/humrep/dead248)
Supplement: dead248_Supplementary_Data_File_S4 [file dead248_supplementary_data_file_s4.docx]

# **Supplementary Data File S4** The R code to identify studies to be excluded according to the exclusion criteria.

**#Animal studies**

Animal<-"[Mm]onkey|[Rr]abbit|[mM]ice|[Rr]ats|[dD]rosophilid｜\\b[Rr]at(s)?\\b|\\b[Pp]ig(s)?\\b|[hH]amsters|[Mm]urine|[mM]ouse|[cC]attle|[pP]rimate|\\b[Cc]ow(s)?\\b|[Ss]heep"

**# Guidelines**

Guidelines<-M %>%

filter(str_detect(Mesh,"Guidelines")==T|str_detect(Mesh,"Advisory Committees")==T|str_detect(Title,"[Bb]est [Pp]ractice")==T|str_detect(Title,"[cC]ommittee|COMMITTEE")==T|str_detect(Title,"[Gg]uideline")==T)

**#Retracted papers**

Retract<-M %>%

filter(str_detect(Abstract,"\\bThis article has been (retracted|withdrawn)")==T|str_detect(Title,"[Rr]etracted|WITHDRAWN")==T| str_detect(Title,"[Cc]ommentary")==T|str_detect(Title,"Erratum"))

**#Case series or case series**

Case_report<- M %>%

filter(str_detect(Title,"[cC]ase report")==T|str_detect(Title,"[Cc]ase series")==T | str_detect(StudyType,"Case Reports")==T)

**#Reviews**

Reviews<-M %>%

filter(str_detect(StudyType,"Systematic Review")==FALSE & str_detect(StudyType,"Review")==TRUE)
