# Supplementary material for: Statistical significance and publication reporting bias in abstracts of reproductive medicine studies
Source: Hum Reprod. 2023 Nov 28;39(3):548–58. doi: 10.1093/humrep/dead248 (PMC10905502; doi:10.1093/humrep/dead248)
Supplement: dead248_Supplementary_Data_File_S5 [file dead248_supplementary_data_file_s5.docx]

# **Supplementary Data File S5** The R code to identify P values, confidence intervals and text in describing statistical significance.

**P-values**

Detect_All_P<-"(([oO]dds ratio|[Rr]isk ratio|[Hh]zard ratio|[Rr]elative risk|[Rr]ate ratio|[Aa]OR|OR|[Aa]HR|HR|[Aa]RR|RR|[Aa]RD|RD|[Mm]ean difference|[Mm]edian difference|[Rr]isk [Dd]ifference|[aA]verage [Dd]ifference|[Dd]ifference(s)? in [Rr]isk|WMD|SMD)((?![Pp]).)*)?(\\s|\\(|[:punct:])[Pp]{1}(\\s|-)*(for)?(value|values)?(for)?(\\s)*(=(\\s)?(or)?(\\s)?[<>](\\s)?|[<>](\\s)?(or|OR)?(\\s)?|[:=<>≤≥]|/=|(\\s)?trend(\\s)?|(\\s)?trend(\\;|<|>|≤|≥|\\:|\\=)?|(\\s)?interaction\\s(effect)?(\\s)?(\\;|<|>|≤|≥|\\:|\\=)?|(\\s)?interaction(effect)?(\\s)?(\\;|<|>|≤|≥|\\:|\\=)?|(\\s)?equals|(\\s)?equal|(\\s)?over|(\\s)?over than|(\\s)?less than or equal to|(\\s)?higher than|(\\s)?larger than|(\\s)?greater than|(\\s)?more than|(\\s)?lower than|(\\s)?less than|less than|(\\s)?smaller than|(\\s)?of <)+(\\s)*(NS|not significant|n.s.|N.S.|ns|nonsignificant|non-significant)?(\\s)?((([0-9][\\,|\\.|\\·|•]|[\\,|\\.|\\·|•])(\\s)*[0-9]*)|[0-9]*)((\\%)?|([x|×]? (\\s)*[0-9]*(\\s)*((exp|Exp|E|e)?(\\s)*((\\((\\s)*(-){1}(\\s)*[0-9]+(\\s)*\\))|((\\s)*(-){1}(\\s)*[0-9]+)))?))"

**Confidence intervals**

Only_CI<-"(([oO]dds ratio|[Rr]isk ratio|[Hh]zard ratio|[Rr]elative risk|[Rr]ate ratio|[Aa]OR|OR|[Aa]HR|HR|[Aa]RR|RR|[Aa]RD|RD|[Mm]ean difference|[Mm]edian difference|[Rr]isk [Dd]ifference|[aA]verage [Dd]ifference|[Dd]ifference(s)? in [Rr]isk|WMD|SMD)((?![Pp|OR]).)*)?(95\\s?\\%?)?\\s?([Cc]onfidence [Ii]nterval(\\s[\\(\\[\\{]\\bCI\\b[\\)\\]\\}])?|\\bCI\\b)\\s?[:punct:]?(\\s)?\\=?\\,?\\s?(\\-)?\\d+\\.?\\d*(\\-|\\sto|\\–|\\,|\\;)\\s?(\\-)?\\d+\\.?\\d*"

**Text in describing statistical significance**

Text_dect<-"(([\\w\\s]+)?(\\s)?[sS]ignificant(ly)?\\s[\\w\\s]+)|[\\w\\s]+\\sdiffer(ence|ences|ent)\\s[\\w\\s]+"

M<-cbind(as.character(M1$PMID),str_extract_all(M1$Abstract,Text_dect,simplify = TRUE))

M <- M %>%

filter(str_detect(P_value,"To determine|objective|To assess|aim|investigate|purpose|known|surgical difference")==FALSE)%>%

filter(str_detect(P_value,"\\bTo\\b")==FALSE)
