# Supplementary material for: Statistical significance and publication reporting bias in abstracts of reproductive medicine studies
Source: Hum Reprod. 2023 Nov 28;39(3):548–58. doi: 10.1093/humrep/dead248 (PMC10905502; doi:10.1093/humrep/dead248)
Supplement: dead248_Supplementary_Data_File_S6 [file dead248_supplementary_data_file_s6.docx]

# **Supplementary Data File S6** The R code to identify effect measures

Point_estimate<-c('\\bCrI\\b','\\bCI\\b','\\bCIs\\b','[mM]ean difference','\\bci\\b','\\bcis\\b','\\bcri\\b','Confidence Interval','Posterior Interval','Credibility Interval','Prediction Interval','Credible Interval','Bayesian Credible Interval','Bayesian Interval','Confidence Limit','Uncertainty Interval','Uncertainly Interval','Uncertainty Range', 'Confidence interval','Posterior interval','Credibility interval','Prediction interval','Credible interval','Bayesian credible interval','Bayesian interval','Confidence limit','Uncertainty interval','Uncertainly interval', 'Uncertainty range', 'confidence interval','posterior interval','credibility interval','prediction interval','credible interval','bayesian credible interval','bayesian interval','confidence limit','uncertainty interval','uncertainly interval', 'uncertainty range', '[eE]ffect\\s[sS]ize','[Rr]ange[:punct:]?(\\s)?\\d+','\\b[Rr][Dd]\\b','[Rr]elative\\s[rR]isk','\\bRR\\b','^RR$','[aA]RR','^RRR$','[rR]isk\\s([Dd]ifference|[Rr]atio)','\\b(A|a)?OR\\b','([aA]djusted)?(\\s|\\-)?([Oo]dds\\s[Rr]atio|\\bOR\\b)',"\\baHR\\b","[hH]azard\\s[rR]atio","\\bHR\\b",'rate ratio',"\\(RR\\s","[Gg]lass\\'s\\s\\Δ","[Gg]lass\\'s\\s\\δ","[Gg]lass\\'s\\s[Dd]elta","[Hh]edges(\\')?s\\sg","[Pp]robability of superiority","[pP]robability\\-of\\-superiority","[sS]uperiority\\s[pP]robability","[Cc]liff\\'s\\s[dD]elta","[Pp]earson correlation coefficient","[Pp]earson(\\')?(s)?(\\s)?r","[Pp]earson product(\\-)?moment correlation coefficient","[bB]ivariate [cC]orrelation","[cC]orrelation [cC]oefficient","[Ss]pearman\\'s (ρ|p|P)","[Ss]pearman\\’s ρ","[rR]ho","[tT]au\\(b\\)","[rR]\\([Tt]au\\)","Rpb","Phi(\\-|\\s)coefficient","φ(\\-|\\s)coefficient","Kendall(\\')?s\\s[tT]","Kendall\\’s τ","Kendall(\\')?s\\s[Tt]au","[Pp]earson(\\')?(s)?contingency coefficient","[Cc]ramer(\\')s?[Vv]","[Gg]oodman and [K]kruskal's [lL]ambda","[Gg]oodman and [K]kruskal's\\sλ","[cC]oefficient of [dD]etermination","[Rr](\\s)?(\\-)2","[cC]oefficient of [mM]ultiple [dD]etermination","[Aa]dj(\\-)?[Rr]","[rR](\\-)?\\s[sS]quared","[cC]ohen(\\')?s\\s[ƒfF]","[cC]orrelation\\s[rR]atio\\s[Ss]quared","[eE]ta(\\-)?[sS]quared","\\η[sS]quared","\\ε2","\\ε(\\s)?[Ss]quared", "[sS]quared [cC]anonical [Cc]orrelation [cC]oefficient","[Bb]ayesian")
