# Supplementary material for: Statistical significance and publication reporting bias in abstracts of reproductive medicine studies
Source: Hum Reprod. 2023 Nov 28;39(3):548–58. doi: 10.1093/humrep/dead248 (PMC10905502; doi:10.1093/humrep/dead248)
Supplement: dead248_Supplementary_Data_File_S7 [file dead248_supplementary_data_file_s7.docx]

# **Supplementary Data File S7** Search Strategy for identify basic research.

BasicStudy<-"(?i)\\bmolecular\\b|humans|meiotic|\\bgene\\b|\\bgenes\\b|expression|protein|proteins|spermatozoa|granulosa|seminal|follicular fluid|genomic|microbio|pathway|molecule|spermatogenic|miRNAs|miRNA|DNA|CD44|chemokine receptor|gamete|mutation|cytogenetic|methylation|SNP|polymorphisms|microarrays|spermatogensis|mammal|germinal|\\bCD[0-9]*\\b"

M$StudyDesign<-ifelse(is.na(M$StudyDesign)==TRUE & str_detect(M$Title,BasicStudy)==TRUE,"Basic research",M$StudyDesign)
