# Supplementary material for: Statistical significance and publication reporting bias in abstracts of reproductive medicine studies
Source: Hum Reprod. 2023 Nov 28;39(3):548–58. doi: 10.1093/humrep/dead248 (PMC10905502; doi:10.1093/humrep/dead248)
Supplement: dead248_Supplementary_Data_File_S8 [file dead248_supplementary_data_file_s8.pdf]

OXFORD
human reproduction
Human Reproduction, 2023, 1–1
https://doi.org/10.1093/humrep/dead248
Supplementary Data

Supplementary Data File S8

List of PMIDs (n = 500) that were randomly selected for manual check.

The 200 PMIDs included in the analysis

32862440, 25847078, 9197445, 9215184, 7726246, 2309813, 15846192, 15507978, 14520188, 14520188, 32434001, 32434001, 32434001, 32434001, 23631687, 23631687, 29884724, 29692677, 11527884, 11527884, 11425814, 10920083, 10875864, 10875864, 10875864, 10739830, 10731533, 10548621, 9848295, 9806578, 9740424, 9740424, 9740424, 9740424, 9512238, 9389829, 9308805, 9159436, 9091334, 9021389, 9021354, 9021354, 8981144, 8816612, 8671448, 8671448, 8671448, 8612825, 8774289, 8774279, 8557129, 8557129, 8557129, 8747064, 8567777, 8567777, 8567768, 7593541, 7890066, 7851592, 17053002, 16952503, 16772283, 16753160, 16616748, 16580673, 16580673, 16580673, 16580673, 16580673, 16580673, 16476679, 16361288, 16359960, 16169398, 16169398, 16126756, 16126756, 15950663, 15589851, 15539443, 15520023, 15271869, 15271869, 14556810, 14507840, 12568840, 11821277, 11821277, 19608174, 19589524, 19589524, 19393998, 19202142, 19202142, 19202142, 19171332, 19171332, 19171332, 19171332, 19054777, 19054777, 19054777, 18854404, 18793777, 18420648, 18420648, 18420648, 18420648, 18326517, 18216034, 18206143, 18166182, 18054934, 18054934, 18054934, 17953963, 17624343, 17418829, 17418829, 24067602, 23543384, 23543384, 23543384, 23543384, 23219009, 23127590, 22892419, 22661547, 22313868, 22313868, 22313868, 22313868, 21784735, 21742732, 21561607, 21474126, 21429951, 21429951, 21333986, 21333986, 21296803, 21296803, 21296803, 21296803, 21130987, 21130987, 21130987, 21122845, 20488804, 36112046, 35940929, 35940929, 35595197, 35595197, 35595197, 35595197, 34269389, 34130800, 33745725, 33744926, 33744926, 33744926, 33744926, 33744926, 33744926, 33744926, 33744926, 32907748, 32709382, 32378719, 32106988, 31407797, 31407797, 30826115, 30826115, 30826115, 29659847, 29659847, 29659847, 29659847, 29659847, 29659847, 29659847, 29659847, 29659847, 29659847, 29659826, 29101996, 28077428, 27619768, 27619768, 27619768, 26783243, 26677955, 26141713, 26141713, 26141713, 26072382, 26072382, 26072382, 26072382, 26072382, 26072382, 26072382, 25518976, 25518976, 25450298, 25450298, 25439846, 25439807, 7805897, 7527424, 7527424, 7527424, 8174741, 8253921, 8253921, 8314970, 1426383, 1426383, 1426329, 1447368, 1639988, 1639988, 1936326, 1894017, 1986962, 2121555, 2307247, 15863157, 17343571, 26381201, 26381201, 26381201, 26283224, 26283224, 26283224, 26283224, 26283224, 25939525, 16822512, 11818090, 11818090, 11818090, 34670164, 30824937, 30824937, 30824937, 29126233, 27329547, 27329547, 27329547, 27329547, 27329547, 24994815, 24067621, 24067621, 24067621, 19106114, 15542541, 33308632, 23871271, 23871271, 22678036, 25730225, 25730225, 25730225, 34384445, 34384445, 34384445, 34384445, 34384445, 32404170, 25442239, 24950982, 24950982, 24950982, 24950982, 24950982, 24476504, 24476504, 24476504, 22950645, 21114859, 12694627, 36339248, 36274014, 36274014, 36274014, 36274014, 36274014, 36274014, 33243661, 33243661, 33243661, 33243661, 33243661, 32565229, 31665451, 31665451, 30979610, 30979610, 30979610, 30979610, 30979610, 30979610, 30979610, 30926176, 30314887, 29888739, 29373224, 29373224, 30895233, 28826600, 28826600, 27793549, 27793549, 27793549, 25682303, 25682303, 25682303, 25496836, 24832373, 24365022, 22116069, 22116069, 22056634, 22056634, 21802364, 21493151, 20089454, 20089454, 19400985, 19298729, 19298729, 18983734, 18492370, 17623535, 17623531, 17623531, 17623531, 17169200, 16569327, 16102288, 15901452, 15820044, 12804191, 12470573, 12470548,"

The 300 PMIDs excluded from the analysis

29259429, 8602299, 22827167, 33345890, 26079138, 16443225, 15749483, 22851718, 28366411, 30244153, 10094875, 23113484, 32741623, 32675056, 16101594, 12560278, 1326851, 11531608, 34593324, 12175710, 29655965, 8134069, 1701442, 33496337, 25936239, 18174599, 9757998, 19049990, 11846715, 25189127, 29995741, 21600635, 16803519, 35221133, 10509488, 1459289, 25711677, 8298402, 31277768, 16681792, 34237463, 2140242, 25323971, 16482612, 20347308, 29395094, 1755467, 23639121, 36154762, 19409550, 16725287, 12576243, 29914564, 7705679, 10472865, 26975901, 26858235, 25747494, 17362718, 23782294, 27890646, 15207682, 20655531, 12797181, 18331737, 21707553, 17156828, 23084567, 27421753, 11004363, 11005126, 29644632, 2316574, 25971554, 10528000, 12388981, 7962420, 25846712, 10593364, 22381597, 11675468, 11972408, 30278175, 32996648, 26726121, 12892685, 20226398, 1977642, 15123057, 28375930, 21897267, 27678886, 7778640, 14607592, 31917139, 11578411, 17337456, 9540928, 27679048, 7693095, 7660774, 30527853, 32925615, 35747402, 11844213, 28238563, 10419730, 8090392, 30129447, 14751164, 9540946, 16626612, 20494220, 30503759, 14675309, 9591497, 28760629, 16113040, 35257371, 26070225, 23812447, 24373607, 17980879, 30833434, 32044310, 22880883, 9932563, 11531279, 35405331, 17316648, 17030354, 12893181, 1689792, 2403173, 10525373, 11532484, 7907045, 11597625, 31553843, 29370405, 32048426, 19406035, 1986618, 8671235, 28456617, 31787144, 7657758, 7926143, 9396911, 20231871, 2245843, 27029748, 16260232, 22975362, 15603571, 12861188, 10561663, 32920856, 24290580, 15760961, 10486503, 32418309, 31792973, 8677125, 12100807, 25753566, 11576257, 9755423, 15467564, 25611633, 20385495, 26593970, 7989514, 34081895, 14599877, 25772774, 31954462, 9389630, 15922981, 26299773, 26226551, 1738516, 17601920, 30710365, 18172174, 24413231, 35712974, 28508540, 33722360, 21605860, 1675344, 16824462, 22349796, 17618753, 10831348, 18554828, 1670709, 10335814, 20184570, 8538484, 17042035, 9358003, 17434502, 28383770, 35320887, 29305255, 36369183, 10506031, 14998943, 17343575, 7705688, 24497418, 2147736, 26164536, 32354468, 8141225, 27648647, 31778255, 35450773, 32043575, 2216268, 24768477, 33726897, 15581986, 18336401, 31228878, 8903916, 21734617, 17826411, 1710520, 8893674, 21895614, 15823112, 12528166, 17617194, 16624390, 33451595, 31927045, 34915539, 1521641, 33168216, 8384706, 8042455, 21489521, 20569835, 12388995, 12965133, 25451694, 17066500, 27789084, 17291236, 18720045, 31472758, 15215487, 23558051, 14662224, 15721421, 27466209, 14768069, 32912616, 15099977, 15304171, 12712098, 9777861, 24736417, 19041243, 22687365, 24100512, 17545686, 12513856, 23422899, 27678776, 15136121, 20382782, 34874585, 10967011, 11209176, 7845152, 19093235, 17187946, 10920317, 16448873, 33349258, 7901087, 2309836, 34674890, 17466048, 24463655, 27105700, 11518891, 9721781, 7540568, 21337658, 1407901, 2037101, 1309944, 23635667, 21047636, 11576279, 22818875
