# Supplementary material for: Statistical significance and publication reporting bias in abstracts of reproductive medicine studies
Source: Hum Reprod. 2023 Nov 28;39(3):548–58. doi: 10.1093/humrep/dead248 (PMC10905502; doi:10.1093/humrep/dead248)
Supplement: dead248_Supplementary_Data_File_S9 [file dead248_supplementary_data_file_s9.pdf]

OXFORD
human reproduction
Human Reproduction, 2023, 1–1
https://doi.org/10.1093/humrep/dead248
Supplementary Data

Supplementary Data File S9

Inferred *P*-values versus directly reported *P*-values.

There were 1334 pairs of *P*-values and confidence intervals extracted from 579 articles. Of the 1334 statements, 1295 (97.1%) of them have a consistent conclusion between inferred *P*-values and directly reported *P*-values while 39 statements do not. We manually checked all the 39 statements and no data extraction error was found. Of the 39 statements, 35 (89.7%) of them shown to be statistically significant in directly reported *P*-values but not inferred *P*-value.
