# Supplementary material for: Statistical significance and publication reporting bias in abstracts of reproductive medicine studies
Source: Hum Reprod. 2023 Nov 28;39(3):548–58. doi: 10.1093/humrep/dead248 (PMC10905502; doi:10.1093/humrep/dead248)
Supplement: dead248_Supplementary_Data_File_S10 [file dead248_supplementary_data_file_s10.pdf]

OXFORD
human reproduction
Human Reproduction, 2023, 1–1
https://doi.org/10.1093/humrep/dead248
Supplementary Data

Supplementary Data File S10

Results of manual check.

Manual check for the automated exclusion and inclusion

Of the 300 articles excluded by automation, none of the excluded articles were identified that should have been included. Of the 200 articles included, 9 (4.5%) articles should have been excluded. Of the nine articles mistakenly included, three were gynaecological articles whereas the other six were reviews or opinion papers.

Manual check for automated basic studies labelling

Of 200 abstracts randomly selected, 23 were identified automatically to be basic studies, and all of them were manfully verified to be basic studies (no false positives). Of 177 articles automatically labelled as non-basic studies, 9 (5.1%) were found to be basic studies (false negatives).

P-values manual check

Among 200 randomly selected abstracts, 155 articles were identified automatically to be free of *P*-values and 2 had been missed (false negative). Among 45 articles that had been automatically detected to have *P*-values, manual verification confirmed none of the articles were *P*-value absent (no false positive).

Of the 149 *P*-values reported by the automated extraction, 2 *P*-values were a statistical threshold applied in the methods of the paper; thus, the specificity was 98.7%. Among 154 *P*-values manually identified, 147 were identified by automated extraction, and 7 *P*-values were missed; thus, the sensitivity was 95.5%. The missed *P*-values were expressed as 'All $P < 0.05$', whereas only one *P* was extracted.

Confidence interval manual check

Among 180 abstracts automatically detected as free of the confidence interval, 10 had been missed (false negatives). Among 20 articles identified to have at least one confidence interval automatically, none of the articles were *P*-value absent (no false positive).

Of the 50 confidence intervals reported by the automated extraction, all of them were found to have confidence intervals, thus the specificity was 100%. Among 79 confidence intervals manually identified, 50 were identified by automated extraction and 29 were missed; thus, the sensitivity was 63.2%. The missed confidence intervals were due to the word 'OR' being separated by multiple words from numbers. An example was 'The OR for AMH as a predictor of implantation in women with unspecified ovarian reserve (n = 1591) was 1.83 (95% confidence interval [CI] 1.49-2.25)'. Among the 29 missed confidence interval, 14 was not statistically significant and 15 was statistically significant.

Statistical significant texts manual check

Among 88 abstracts that are automatically labelled as to be free of text in describing significance, 11 had been missed (false negative). Among 46 abstracts that were automatically reported to have at least one significance in text, none of them were absent from significance (no false positive).

Of the 78 significant texts reported by the automated extraction, 8 were not meant for the significance of the findings, thus the specificity was 90.0%. They either meant for the statistical significance threshold or were describing the significance at the baseline level. Among 104 significant texts manually identified, 70 were identified by automated extraction and 34 were missed, thus the sensitivity was 67.3%. The missed statistical significance texts were because only one word of significance was used to describe multiple variables. For example: 'Mini-Percoll produced specimens with significantly greater normal sperm morphology, morphology improvement, motility, hypoosmotic swelling, and survival than obtained with the other two separation methods'.

The proportion of at least one significant findings revealed by manual check

To confirm if the proportion of abstracts reporting at least one confidence interval or significant term via automated extraction is a reliable estimate, we manually checked the 200 sampled abstracts to calculate how many of them contained at least one statistically significant finding in the form of confidence intervals or texts.

Among 30 articles reporting confidence intervals identified by manual check, 83.3% (25/30) of them contained at least one statistically significant confidence interval. Similarly, among 57 articles that did not report *P*-values or confidence intervals but reported significant terms, 80.7% (11/57) of them contained at least one statistically significant term.

A summary table of the sensitivity and specificity of the automated data extraction

	Sensitivity (%)	Specificity (%)
Basic studies classification	94.9	100
Confidence intervals	63.2	100
P-values	98.7	95.5
Statistical significant texts	67.3	90.0
