# Supplementary figures and images for: Statistical significance and publication reporting bias in abstracts of reproductive medicine studies
Source: Hum Reprod. 2023 Nov 28;39(3):548–58. doi: 10.1093/humrep/dead248 (PMC10905502; doi:10.1093/humrep/dead248)

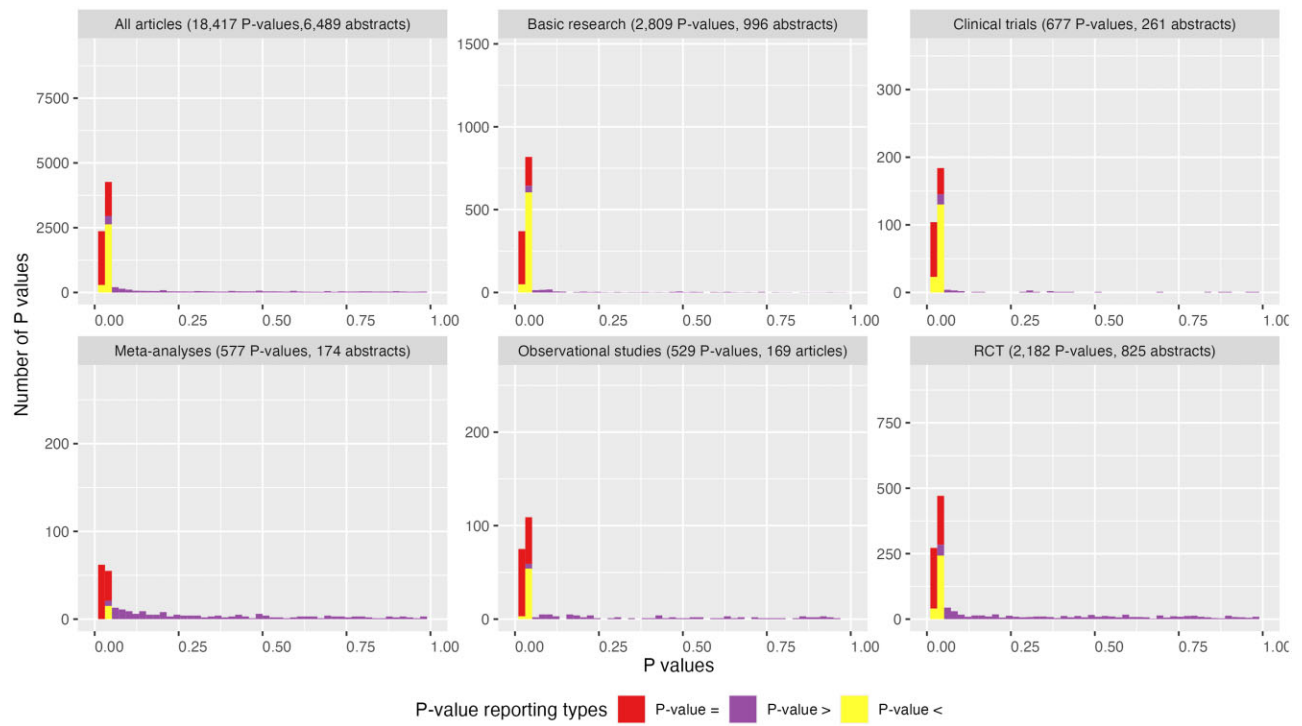

**Supplementary Figure S3.** The full spectrum of the extracted P-values.

Supplement: dead248_Supplementary_Figure_S3 [file dead248_supplementary_figure_s3.pdf]
