# Supplementary material for: Statistical significance and publication reporting bias in abstracts of reproductive medicine studies
Source: Hum Reprod. 2023 Nov 28;39(3):548–58. doi: 10.1093/humrep/dead248 (PMC10905502; doi:10.1093/humrep/dead248)
Supplement: dead248_Supplementary_Figure_S4 [file dead248_supplementary_figure_s4.pdf]

OXFORD
human reproduction
Human Reproduction, 2023, 1–1
https://doi.org/10.1093/humrep/dead248
Supplementary Data

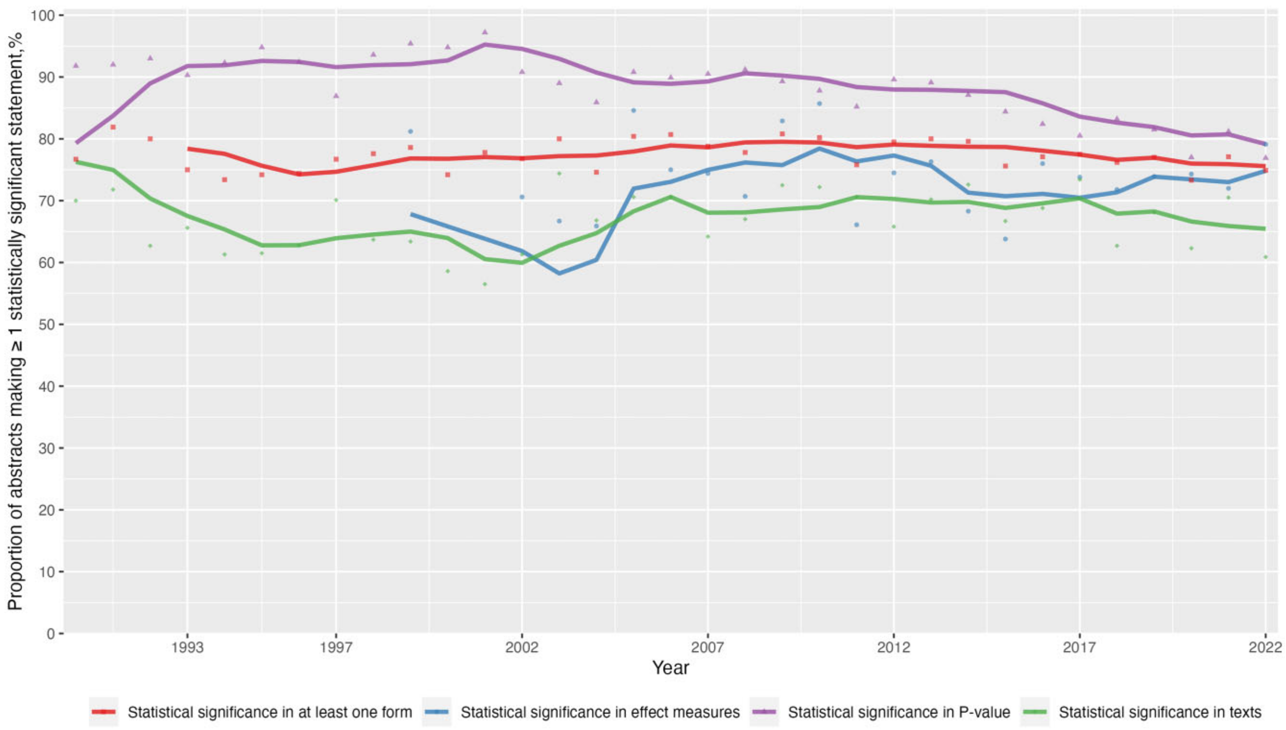

Supplementary Figure S4. Frequency of abstracts making ≥1 statistically significant statement among abstracts making at least one statistical inference by study design (texts ambiguous in their statistical significance were excluded).
