# Supplementary material for: Statistical significance and publication reporting bias in abstracts of reproductive medicine studies
Source: Hum Reprod. 2023 Nov 28;39(3):548–58. doi: 10.1093/humrep/dead248 (PMC10905502; doi:10.1093/humrep/dead248)
Supplement: dead248_Supplementary_Table_S1 [file dead248_supplementary_table_s1.pdf]

OXFORD
human reproduction
Human Reproduction, 2023, 1–1
https://doi.org/10.1093/humrep/dead248
Supplementary Data

Supplementary Table S1. The list of Q1 journals indexed in Journal Citation Reports 2021 under the category of Reproductive Biology (n = 7) and Obstetrics and Gynaecology (n = 16).

Journal name	Category	JIF quartile	JIF
Human Reproduction Update	Obstetrics and Gynaecology—SCIE	Q1	17.2
American Journal of Obstetrics and Gynecology	Obstetrics and Gynaecology—SCIE	Q1	10.7
American Journal of Obstetrics & Gynecology MFM	Obstetrics and Gynaecology—SCIE	Q1	8.7
Ultrasound in Obstetrics & Gynecology	Obstetrics and Gynaecology—SCIE	Q1	8.7
Obstetrics and Gynecology	Obstetrics and Gynaecology—SCIE	Q1	7.6
Fertility and Sterility	Obstetrics and Gynaecology—SCIE	Q1	7.5
BJOG-an International Journal of Obstetrics and Gynaecology	Obstetrics and Gynaecology—SCIE	Q1	7.3
Human Reproduction Open	Obstetrics and Gynaecology—SCIE	Q1	7.1
Human Reproduction	Obstetrics and Gynaecology—SCIE	Q1	6.4
Gynecologic Oncology	Obstetrics and Gynaecology—SCIE	Q1	5.3
Maturitas	Obstetrics and Gynaecology—SCIE	Q1	5.1
Journal of Gynecologic Oncology	Obstetrics and Gynaecology—SCIE	Q1	4.8
International Journal of Gynecological Cancer	Obstetrics and Gynaecology—SCIE	Q1	4.7
Reproductive Biomedicine Online	Obstetrics and Gynaecology—SCIE	Q1	4.6
Acta Obstetricia et Gynecologica Scandinavica	Obstetrics and Gynaecology—SCIE	Q1	4.5
Molecular Human Reproduction	Obstetrics and Gynaecology—SCIE	Q1	4.5
International Journal of Gynecology & Obstetrics	Obstetrics and Gynaecology—SCIE	Q1	4.4
Journal of Minimally Invasive Gynecology	Obstetrics and Gynaecology—SCIE	Q1	4.3
Best Practice & Research Clinical Obstetrics & Gynaecology	Obstetrics and Gynaecology—SCIE	Q1	4.3
Breast	Obstetrics and Gynaecology—SCIE	Q1	4.3
Reproductive Medicine and Biology	Obstetrics and Gynaecology—SCIE	Q1	4.0
Human Reproduction Update	Reproductive Biology—SCIE	Q1	17.2
Fertility and Sterility	Reproductive Biology—SCIE	Q1	7.5
Human Reproduction Open	Reproductive Biology—SCIE	Q1	7.1
Human Reproduction	Reproductive Biology—SCIE	Q1	6.4
Journal of Ovarian Research	Reproductive Biology—SCIE	Q1	5.5
Reproductive Biology and Endocrinology	Reproductive Biology—SCIE	Q1	5.0
Reproductive Biomedicine Online	Reproductive Biology—SCIE	Q1	4.6

JIF, journal impact factor.
